# Stress-Coping Humoral Glycolipids Produced by Mice Given Controlled Bathing Treatments

**DOI:** 10.1155/2019/4972186

**Published:** 2019-11-03

**Authors:** Yutaka Masuda, Hiroto Narita, Hiroaki Hasegawa

**Affiliations:** ^1^Psychosomatic Division, Graduate School of Medicine, Akita University, Hondo 1-1-1, Akita 010-8543, Japan; ^2^Department Mechanical and Intelligent Engineering, School of Engineering, Utsunomiya University, Youtou 7-1-2, Utsunomiya 321-8585, Japan

## Abstract

Mammalians have recognition-behavioral stress-coping neuronal module system followed by some humoral glycolipids. A sulfated Galbeta1-4GlcNAc-lipid promotes the serotonergic module regulating the emotional behaviors for not-wasting the physical strength; GalNAcalpha1-3GalNAc-lipid promotes the adrenergic module inducing the behaviors escaping from the uneasy situation, and sulfated Fucalpha1-2Gal-lipid protects the cholinergic module keeping the stressor-memory from the ischemia-stress. Mouse given bathing recognizes the stressors to be coped with in the treatment. We previously observed mouse given CO_2_-microbubble-bathing increased the behavior escaping from the bathing situation. Mouse given CO_2_-microbubble-bathing would recognize the other stressors to be coped with in the treatment. We examined stress-coping glycolipids produced by mice given controlled bathing treatments, and got the following results. A sulfated Galbeta1-4GlcNAc-lipid production was increased by the acidic bathing condition and the dissolved CO_2_, GalNAcalpha1-3GalNAc-lipid production was increased by the dissolved CO_2_, and sulfated Fucalpha1-2Gal-lipid production was increased by the acidic bathing condition. We understood the mice treated with CO_2_-microbubble-bathing would recognize the acidic bathing condition and the dissolved CO_2_, but not the microbubble, as the other stressors to be coped.

## 1. Introduction

### 1.1. Background

We have investigated mammalian recognition-behavioral stress-coping system. We previously found mouse given CO_2_-microbubble-bathing showed the behavior escaping from the bathing situation, more than mouse given bathing did [1].

### 1.2. Theory

Mammalian brains work via network of the functional neuronal modules [2]. We found some humoral glycolipids following the recognition-behavioral stress-coping modules. A sulfated Galbeta1-4GlcNAc-lipid (3-O-Sulfo-Beta-D-Galactosyl-(1->4)-N-Acetyl-Beta-D-Glucosamine-lipid; sG1-4GN) promotes the serotonergic module regulating the emotional behaviors for not-wasting the physical strength [3, 4]. A GalNAcalpha1-3GalNAc-lipid (GalNAcalpha1->3GalNAc-lipid; GN1-3GN) promotes the adrenergic module inducing the behaviors escaping from the uneasy situation [5, 6]. And sulfated Fucalpha1-2Gal-lipid (Fucalpha1-2[6OSO3]Galbeta1-4Glcbeta-lipid; sF1-2G) protects the cholinergic module keeping the stressor-memory from the ischemia-stress, as an adaptogen did [4, 7, 8]. Mammalians produce the stress-coping glycolipids corresponding to the stressors.

### 1.3. Hypothesis

Mouse given bathing recognizes the stressors to be coped in the treatment. We hypothesized mouse given CO_2_-microbubble-bathing would recognize the other stressors to be coped with in the treatment. In the present study, we examine the stress-coping glycolipids produced by mice given controlled bathing treatments.

## 2. Materials and Methods

### 2.1. Animals

Female 9-weeks-old DDY mice were purchased from SLC Co. (Hamamatsu, Japan) for using in the present study. All experiments were conditioned in accordance with animal research regulations at Akita University School of Medicine (the approval number: a-1-2824).

### 2.2. Bathing Apparatus

A water-tank (the diameter 40 cm, the depth 70 cm) was prepared at room temperature (RT). A 20°C tap-water was poured in the tank to 50 cm depth. A microbubble-generator and absorb pipe were settled at a depth of the tank, and an electric pump and a CO_2_-cylinder were settled outside of the tank.

### 2.3. Bathing pH Condition and Microbubble-Generator

A bath salt generally marketed was prepared for keeping pH 5 bathing condition. Slit-type microbubble-generator was prepared. The generator produces microbubble by passing-through high-speed water-flow containing gas [9].

### 2.4. Bathing Procedure

Mice were treated with bathing in the water-tank filled with the tap-water for 3 min as pre-treatment. One day after this, 6 mice were treated with the tap-water bathing for 3 min (Bathing group: B group) as Positive Control, another 6 mice were treated with the tap-water bathing in pH 5 bathing condition for 3 min (pH condition group: PH group), another 6 mice were treated with the tap-water bathing generated air-microbubble for 3 min (Microbubble-bathing group: MB group), and the other 6 mice were treated with the tap-water bathing generated CO_2_-microbubble for 3 min (CO_2_-microbubble-bathing group: CM group). Immediately after these treatments, the mice were sacrificed by the neck-location, and blood was collected. The sera were pooled and restored at 4°C.

### 2.5. Humoral Lipid Fractionation

Humoral lipid fractionation was performed as previously described [4]. Briefly, 1.25 ml of chloroform and 2.5 ml of methanol were added to each 1 ml of the pooled serum. The solution was intensively mixed for 3 min and incubated for 10 min at RT. Then, 1.25 ml of chloroform was added to the solution, and followed by intensive mixing for 30 s. A 1 ml of water was added to the solution, and followed by intensive mixing for another 30 s. The mixture was then centrifuged at 150 gravitudes for 10 min at RT. The lower chloroform layer was collected, and the solvent chloroform was evaporated at RT. The extracted lipids were then suspended in 1 ml of water. The solution was applied to 0.5 ml of an ion exchanger DE-52 (Whatman Co., Maidstone, UK) column, which had been saturated with 10 mM NaHCO_3_, pH 8.3, and washed with water. Samples were eluted with 0.5 ml consecutive washes of 50, 100, 150, 200, 250, and 300 mM NaCl. Fractions eluted with 100, 150, and 250 mM NaCl were then diluted to 1 ml with water as the present samples.

### 2.6. Sulfate-Radical Elimination

Stress-coping humoral glycolipids fractionated with 100 mM NaCl and 250 mM NaCl are sulfated. Sulfate-radical was eliminated from the glycolipids for measuring the terminal sugar-chain reactivity as previously described [4]. Briefly, lipids were extracted again from 800 *µ*l of the sample solutions by using methanol-chloroform method as described above. To the extracted lipids were added 400 *µ*l of the reagent containing silyl-agents of TMS-HT kit (Tokyo Chemical Industry Co. Tokyo, Japan), and then, incubated at 90°C for 3 h. To the solution was added 800 *µ*l of water, and intensively mixed for 30 s.

### 2.7. Measurement of the Glycolipid Production

Bipolar glycolipids attach to a plastic plate in 50% ethanol solution. A modified Enzyme-Linked ImmunoSorbent Assay (ELISA) was performed for measuring the glycolipid production as previously described [4]. Briefly, the sample solutions eluted with 150 mM NaCl, the sulfate radical-eliminated sample solutions fractionated with 100 mM NaCl and 250 mM NaCl, or physiological saline (PS) as Negative Control, was prepared to 50% ethanol solution. A 100 *µ*l of the solution was poured into a well of a 96-well plastic plate (Sumitomo-Bakelite Co., Tokyo, Japan). ELISA was performed with the use of 300 *µ*l of 5% bovine serum albumin (Sigma-Aldrich Co., St. Louis, MO, USA) as a blocker, a biotinized-lectin of Recinus communis recognizing Galbeta1-4GlcNAc, that of Dolichos biflorus recognizing GalNAcalpha1-3GalNAc or that of Aleuria aurentia recognizing Fucalpha1-2Glc (Seikagaku Co., Tokyo, Japan), peroxidase-conjugated-avidin (Seikagaku Co.), and the coloring kit (Sumitomo Bakelite Co.). Then, the light absorbance was measured at the dual wavelength of 450/655 nm. The ELISA procedure was individually performed on 5 different plates.

### 2.8. Statistical Analyses

Mann-Whitney *U*-test was used for finding difference from Positive Control. A *p* < 0.05 (*n*_1_ = *n*_2_ = 5) was considered as a significant difference.

## 3. Results

### 3.1. sG1-4GN Production

A sG1-4GN is produced in the fraction eluted with 100 mM NaCl. The glycolipid production was detected in all of the samples. The PH group mice increased the glycolipid production, and the CM group mice further increased the glycolipid production (Table 1).

### 3.2. GN1-3GN Production

A GN1-3GN is produced in the fraction eluted with 150 mM NaCl. The glycolipid production was detected in all of the samples. The CM group mice increased the glycolipid production (Table 2).

### 3.3. sF1-2G Production

A sF1-2G is produced in the fraction eluted with 250 mM NaCl. The glycolipid production was detected in all of the samples. The PH group mice increased the glycolipid production (Table 3).

## 4. Discussion

A sG1-4GN is produced for maintaining the physical strength, GN1-3GN is produced for inducing the behaviors escaping from the uneasy situation, and sF1-2G is produced for keeping the stressor-memory. In the present study, the B group mice produced sG1-4GN, GN1-3GN and sF1-2G. This suggests the tap-water bathing gave a stressor depriving of the physical strength, an uneasiness to be avoided, and a stressor to be memorized, to the mice. The PH group mice and the CM group mice, but not the MB group mice, increased sG1-4GN production. Furthermore, the production of the CM group mice was larger than that of the PH group mice. These suggest the mice recognized the acidic bathing condition as another stressor depriving of the physical strength, and did the dissolved CO_2_ as the other stressor depriving of the physical strength. The CM group mice, but not the PH group mice nor the MB group mice, increased GN1-3GN production. This suggests the mice recognized the dissolved CO_2_ as an uneasy situation factor to be avoided. The PH group mice, but not the MB group mice nor the CB group mice, increased sF1-2G production. This suggests the mice recognized the acidic bathing condition, but not the dissolved CO_2_, as a stressor to be memorized.

Mouse senses stimulations, recognizes the stimulations as stressors, and produces the stress-coping glycolipids corresponding to the stressors. Mice given the presented bathing treatments would sense the stimulations mainly via the skin. Wet fur of the mice would block mechanical stimulation of microbubble, nevertheless, the microbubble makes CO_2_-dissolution easy. Dissolved CO_2_ induces acidic bathing condition, however, the mice recognized the dissolved CO_2_ as a stressor different from the acidic bathing condition. A rabbit treated with bathing applied CO_2_ elevates the subcutaneous tissue PO_2 _[10]. The mice might recognize the PO_2 _elevation as a stressor depriving of the physical strength and an uneasy stimulation to be avoided. As the PO_2_ elevation frequently occurs, the dissolved CO_2_ might not be recognized as a stressor to be memorized.

A strong stress induces secretions of gene-expressing hormones via Hypothalamus-Pituitary Axis. The stress-coping glycolipids would be produced via the hormone-secretion. Furthermore, these glycolipids may be cerebrosides [4]. Ganglioside, one of cerebrosides, increases synaptic plasticity in the hippocampus [11]. The presented stress-coping glycolipids might also promote synaptic plasticity in the corresponding neuronal modules. On the other hand, the skin also produces ceramide, lipid portion of cerebrosides [12]. The stress-coping glycolipids might be produced not only in the brain but also in the skin, nevertheless, mechanism of the glycolipids produced in the peripheral blood is not clarified in the present time.

## 5. Conclusion

Mouse has the recognition-behavioral stress-coping system followed by some humoral glycolipids. We investigated the stress-coping glycolipids produced by mice given controlled bathing treatments, and found the mice recognized the dissolved CO_2_ and the acidic bathing condition as the different stressors to be coped.

## Figures and Tables

**Table 1:** The mean ± SD of sG1-4GN reactivity in the samples obtained from mice given controlled bathing treatments.

	Light absorbance (450/655 nm)
Positive control: B group (given tap-water bathing)	0.119 ± 0.011
PH group (given pH 5 bathing)	^∗^0.157 ± 0.004
MB group (given air-microbubble-bathing)	0.118 ± 0.017
CM group (given CO_2_-microbubble-bathing)	^∗^0.291 ± 0.014
Negative control (the sample: physiological saline)	0.051 ± 0.007

The sG1-4GN: sulfated Galbeta1-4GlcNAc-lipid promoting the serotonergic module regulating the emotional behaviors for not-wasting the physical strength.

^∗^ *p* < 0.05 compared to positive control, Mann-Whitney *U* test (*n*_1_ = *n*_2_ = 5).

**Table 2:** The mean ± SD of GN1-3GN reactivity in the samples obtained from mice given controlled bathing treatments.

	Light absorbance (450/655 nm)
Positive control: B group (given tap-water bathing)	0.138 ± 0.010
PH group (given pH 5 bathing)	^∗^0.148 ± 0.024
MB group (given air-microbubble-bathing)	0.138 ± 0.020
CM group (given CO_2_-microbubble-bathing)	^∗^0.230 ± 0.017
Negative control (the sample: physiological saline)	0.053 ± 0.010

The GN1-3GN: GalNAcalpha1-3GalNAc-lipid promoting the adrenergic module inducing the behaviors escaping from the uneasy situation.

^∗^ *p* < 0.05 compared to positive control, Mann-Whitney *U* test (*n*_1_ = *n*_2_ = 5).

**Table 3:** The mean ± SD of sF1-2G reactivity in the samples obtained from mice given controlled bathing treatments.

	Light absorbance (450/655 nm)
Positive control: B group (given tap-water bathing)	0.098 ± 0.008
PH group (given pH 5 bathing)	^∗^0.139 ± 0.021
MB group (given air-microbubble-bathing)	0.089 ± 0.007
CM group (given CO_2_-microbubble-bathing)	0.101 ± 0.016
Negative control (the sample: physiological saline)	0.059 ± 0.016

The sF1-2G: sulfated Fucalpha1-2Gal-lipid protecting the cholinergic module keeping the stressor-memory from the ischemia-stress.

^∗^ *p* < 0.05 compared to positive control, Mann-Whitney *U* test (*n*_1_ = *n*_2_ = 5).

## Data Availability

PubChem compounds is available for understanding structures of the stress-coping humoral glycolipids.

## Supplementary Materials

Supplementary Material Raw data of light absorbance indicating the glycolipid production.
